# The economic impact of caregiving for individuals with Angelman syndrome in the United States: results from a caregiver survey

**DOI:** 10.1186/s13023-025-03551-4

**Published:** 2025-02-21

**Authors:** John Jarvis, Elizabeth Chertavian, Marric Buessing, Taylor Renteria, Lufei Tu, Lauren Hoffer, Ryan Fischer, Amanda Moore, Meagan Cross, Megan Tones

**Affiliations:** 1 Medicus Economics, LLC, 2 Stonehill Ln, Milton, MA 02186 USA; 2 Foundation for Angelman Syndrome Therapeutics, Austin, TX USA; 3 Angelman Syndrome Foundation, Aurora, IL USA; 4 Foundation for Angelman Syndrome Therapeutics, Cairns, QLD Australia; 5 Office of eResearch, Queensland University of Technology, Brisbane, QLD Australia

**Keywords:** Angelman syndrome, Caregiver, Caregiver burden, Work productivity, Economic burden

## Abstract

**Background:**

Angelman syndrome (AS) is a rare neurogenetic disorder characterized by persistent cognitive and functional impairments that necessitate lifelong care. Caring for individuals with AS leads to substantial household costs, as well as impacts on work productivity, leisure time, and quality of life for caregivers. The economic value of these impacts in the United States (US) has not been well studied. We conducted a survey of US caregivers for persons with AS to quantify the annual economic impact of caregiving. Information on AS-related economic impacts was gathered, including household costs, employment impacts, leisure time loss, and caregiver healthcare costs. The survey did not gather information on direct medical care costs borne by healthcare insurers or other economic impacts to the US government and other stakeholders.

**Results:**

A total of 105 caregivers completed the survey and 105 individuals with AS were represented. Most caregivers were female (89.5%), white (83.8%), and identified as the primary caregiver (75.2%). Most individuals with AS represented in the sample were age < 18 (82.9%). The annual economic impact of caregiving for persons with AS averaged $79,837 (SD $55,505). Costs related to employment impacts and lost work productivity in the past 12 months accounted for most (53%) of this impact and averaged $42,697 (SD $28,309). Household costs incurred in the past 12 months for goods and services to better accommodate individuals with AS were $29,680 (SD $47,753). Leading contributors included vehicle purchases and modifications (mean $6,717; SD $17,791), professional caregiving (mean $6,123; SD $17,335), home modifications and repairs (mean $4,387; SD $15,734), and supportive therapy (mean $3,269; SD $7,564). Economic impacts in the past 12 months from lost leisure time and incremental healthcare costs for caregivers were estimated to be $6,634 (SD $4,652) and $827 (SD $2,072), respectively.

**Conclusions:**

Caregivers incur substantial costs to accommodate individuals with AS, as well as substantial impacts related to employment and leisure time. This study’s findings may be utilized in future research to better estimate the value from therapeutic advances in AS and direct resources toward mitigating economic impacts for households.

**Supplementary Information:**

The online version contains supplementary material available at 10.1186/s13023-025-03551-4.

## Background

Angelman syndrome (AS) is a rare neurogenetic disorder with an estimated prevalence of 1 in ~ 12,000 to ~ 20,000 [1, 2]. AS is caused by loss of function of the ubiquitin-protein ligase E3A (*UBE3A*) gene, which is critical for synaptic development and neural plasticity, as well as other functions [3]. As such, AS is characterized by cognitive disability, global developmental delays, communication impairment, absent or near-absent speech, seizures, motor deficits, ataxia, sleep disturbances, gastrointestinal issues, and behavioral impacts such as anxiety and hyperactivity [1–5]. Due to the persistent nature of these impairments, individuals with AS typically require high levels of support and supervision throughout their lifetime. Many individuals with AS continue to live in their parents’ homes throughout adulthood, while some move into group homes or residential centers [6, 7].

Given the high level of support needed, caring for individuals with AS can have a considerable impact on families. Caregiving duties, often provided by parents, can compete for time with other tasks, such as chores, errands, family activities, and personal leisure [8–10]. Continuous management of seizures and sleep disturbances can also contribute to physical impairments for caregivers, as well as fatigue, stress, fear, anxiety, and depression [8–11]. These impacts can extend to the workplace by lowering productivity, requiring reduced working hours to make time for caregiving duties, or resulting in early retirement [8, 9]. Households may also incur expenses to accommodate functional and cognitive impairments for individual with AS, such as modifications to make homes or vehicles safer or more accessible, paying for at-home professional caregiving, or paying for supportive goods or therapies (e.g., occupational therapy).

While previous research has provided a framework for understanding the caregiver and family impacts of AS, the extent of these impacts have not been quantified in the United States (US) [4, 12]. Several recent studies have assessed the caregiver impacts of AS in Australia [13, 14]. Hartmanis et al. (2023) estimated that caring for a person with AS would result in a loss of 38% in productivity-adjusted life years over a ten-year period [13]. Separately, Baker et al. (2021) found that the annual costs of raising a child with AS were two to three times higher than the costs of raising a child with other rare disorders [14]. While these studies demonstrate significant caregiving impacts associated with AS, international differences in work cultures, price levels, and public support services make it difficult to generalize these findings to US caregivers [14–16]. As such, the objective of this study was to quantify the economic impacts of caring for an individual with AS to family caregivers in the US. Authors anticipated that the economic impact to US caregivers would be substantial given these prior ex-US research findings, but did not have a hypothesis as to whether and how this impact might differ for US caregivers.

## Methods

### Study design

We conducted a cross-sectional online survey of adult family caregivers for individuals with AS residing in the US. Caregivers were asked about several areas of life that could be impacted by caregiving, including household expenses related to accommodations or care, healthcare costs for caregivers, employment-related impacts, lost leisure time, and quality of life impacts. Responses were used to estimate the annual economic cost of these impacts to caregivers. The survey did not gather information on direct medical care costs borne by healthcare insurers or other economic impacts to the US government and other stakeholders.

### Survey development

The survey was developed with input from the Foundation for Angelman Therapeutics (FAST), the Angelman Syndrome Foundation (ASF), caregivers of individuals with AS, academic clinical experts, and representatives from pharmaceutical and biotechnology companies with AS therapies in development [17, 18]. Both qualitative and quantitative piloting was conducted with caregivers to develop the survey instrument and confirm instrument validity.

### Data collection

The survey was embedded as a module within the Global Angelman Syndrome Registry (GASR), an internet-based disease registry that collects data from parents and caregivers regarding the natural history and management of AS [19, 20]. For individuals with AS represented in the survey, some demographic and clinical data (e.g., AS genotype) were gathered from the GASR database. The design and implementation of the module within the GASR database was supported by the GASR Governance Board. Ethical approval to conduct this study was provided by Mater Research and the GASR Governance Board, which oversees research access to de-identified registry data. Informed consent was obtained from all caregivers prior to initiation of the survey.

### Survey recruitment

Survey respondents were recruited through networks maintained by FAST and ASF. The survey sample was restricted to individuals who voluntarily cared for an individual with AS for the past twelve months, could provide estimates of the costs and impacts of the care they provided, were age ≥ 18 years, resided in the US, and were registered in the GASR during the study recruitment period (December 2022 through May 2023). To be included in the analytical sample, caregivers were required to complete the survey, defined as answering the final survey question. Outreach was conducted through the GASR database to encourage caregivers with partial survey responses to complete the survey.

### Survey components

#### Demographic and clinical characteristics

Caregivers provided sociodemographic information for themselves and for the person with AS for whom they provided care. Caregivers also provided details regarding their caregiving role and responsibilities, such as their relationship to the person with AS, whether they identified as a primary, secondary, or split-duty caregiver, and the number of years spent providing care. Additionally, they provided details regarding the caregiving needs of the person with AS, including hours per day of care received in the past week, level of professional caregiving needed, and whether they could be left alone for periods of time.

#### Household costs to accommodate and care for individuals with AS

Household costs for AS-related accommodations and care were captured for items in the following categories: home modifications or repairs, vehicle purchases or modifications, medical equipment purchases, long-term care, professional caregiving, supportive therapy, school or educational expenses, and out-of-pocket healthcare expenses (e.g., out-of-pocket costs for outpatient visits or prescription drugs). For each item, caregivers were asked to report their household’s total AS-related expenditures in the past twelve months. Caregivers were asked to provide their best estimate of the costs that were specifically paid by their households (e.g., credit card, cash, family savings), not including any costs paid by external sources (e.g., health insurance, government, or charitable organizations). Caregivers were asked to provide their best estimate of costs that were incurred as a direct result of the care recipient’s AS condition.

#### Caregiver healthcare costs

Caregivers were asked if they had received any pharmaceutical treatments or medical care in the past twelve months as a result of caregiving for a person with AS. If so, caregivers were asked how much they had spent out of pocket over the past twelve months. Caregivers were asked to provide their best estimate of costs that were incurred as a direct result of the care recipient’s AS condition.

#### Caregiver employment-related impact

Caregivers were asked about changes in work status, working hours, or productivity during working hours as a result of caregiving for the person with AS. Questions related to days of missed work and work productivity loss among employed individuals were included from the caregiver version of the Work Productivity and Activity Impairment (WPAI) questionnaire [21].

#### Caregiver leisure time impact

Caregivers were asked to estimate average leisure time loss per week as a result of their caregiving responsibilities. Questions for estimating leisure time loss were derived from the iMTA Valuation of Informal Care Questionnaire (iVICQ) [22].

#### Caregiver quality of life

Caregivers were asked several questions about their own quality of life and how it might be impacted by caregiving. Caregivers provided a self-assessment of their caregiving situation derived from the Care-related Quality of Life instrument (CarerQol), an instrument that is included as part of the iVICQ questionnaire [22]. The choice of quality of life questions was derived based on stakeholder input and qualitative piloting to ensure that quality of life questions were representative of experiences for US caregivers of individuals with AS. The CarerQol instrument has also been used extensively in prior research to assess quality of life impacts for caregivers of other pediatric populations [23–26].

Additional File 1 includes additional information for each category of impact.

### Statistical analyses

#### Demographic and clinical characteristics

Characteristics were summarized separately for caregivers and persons with AS for whom they provided care. Categorical variables were summarized using frequencies and percentages, while continuous variables were summarized using means and standard deviations (SD).

#### Household costs to accommodate and care for individuals with AS

The number and proportion of caregivers reporting an expense in the past twelve months were reported for each cost category. Costs were summarized in terms of means and SDs for each cost category and overall. Additional File 1 includes additional information.

#### Caregiver healthcare costs

The number and proportion of caregivers reporting caregiving-related healthcare expenses in the past twelve months were reported. Costs were summarized for pharmaceutical, medical, and combined healthcare expenses in terms of means and SDs.

#### Caregiver employment-related impact

The annual economic value of lost work productivity was estimated by multiplying the mean (SD) reported weekly hours of lost work productivity by a nationally representative hourly wage ($33.74 as of July 2023) [27], and further multiplying by 52 weeks per year. Calculations were conducted separately for the three components of employment impact due to caregiving demands (early retirement, reduced working hours, reduced work productivity) as well as overall.

#### Caregiver leisure time impact

The annual economic value of lost leisure time was estimated using similar methods to lost work productivity, with each hour of leisure loss valued at 35% of the national hourly wage, consistent with methods from prior caregiver research [28].

These components of caregiver impacts were added together to estimate the total annual economic impact of caregiving associated with AS. Annual caregiver impacts were also descriptively summarized by the age of individuals with AS (0 to 5, 6 to 12, 13 to 17, and 18 +). Other survey outcomes were summarized in terms of proportions and depicted graphically. Additional File 1 contains additional details regarding methods for study calculations.

## Results

### Study sample

A total of 127 surveys were collected from caregivers who met all screening criteria, 22 of which were excluded from the analytical sample due to survey non-completion. The final analytical sample included 105 caregivers for persons with AS, representing 105 individuals with AS receiving care (Tables 1, 2; Supplementary Tables 1a-b, Additional File 1). Mean (SD) caregiver age was 43 (10.5) years. Most caregivers were female (89.5%) and white (83.8%), and most identified as the primary caregiver (75.2%). More than half were employed full time or part time (36.2% and 21.0%, respectively), while 24.8% were homemakers and 7.6% were unemployed. More than two thirds had a Bachelor’s or postgraduate degree (39.0% and 31.4%, respectively).

**Table 1:** Caregiver demographics

Demographic variables	N = 105
Age in years, mean (SD)	43.0 (10.5)
Sex (male), n (%)	11 (10.5%)
Race, n (%)	
Asian	7 (6.7%)
Black or African American	3 (2.9%)
Hispanic or Latino	8 (7.6%)
White	88 (83.8%)
Current work situation, n (%)	
Working full time for pay (employed or self-employed)	38 (36.2%)
Working part time for pay (employed or self-employed)	22 (21.0%)
Student	3 (2.9%)
Unemployed	8 (7.6%)
On long-term sick leave	1 (1.0%)
Retired	3 (2.9%)
Homemaker	26 (24.8%)
Other	4 (3.8%)
Level of education, n (%)	
High school degree	2 (1.9%)
Some college or associate degree	28 (26.7%)
Bachelor’s degree	41 (39.0%)
Postgraduate degree	33 (31.4%)
Other/Not reported	1 (1.0%)
*Caregiving-related variables*	
Relationship to the person with AS, n (%)	
Mother or father	105 (100.0%)
Caregiver responsibilities, n (%)	
Primary caregiver	79 (75.2%)
Secondary caregiver	2 (1.9%)
Evenly splits responsibilities with another household member	24 (22.9%)

*AS* Angelman syndrome; *SD* standard deviation

**Table 2:** Demographic and clinical characteristics of persons with AS

Demographic variables	N = 105
Age in years, n (%)	
0 to 5	32 (30.5%)
6 to 12	40 (38.1%)
13 to 17	15 (14.3%)
18+	18 (17.1%)
Sex (male), n (%)	52 (49.5%)
Race, n (%)	
Asian	8 (7.6%)
Black or African American	5 (4.8%)
Hispanic or Latino	14 (13.3%)
Native Hawaiian or other Pacific Islander	1 (1.0%)
White	90 (85.7%)
*Clinical and caregiving-related variables*	
AS genotype, n (%)	
Deletion	51 (48.6%)
Mutation	26 (24.8%)
Imprinting center defect	2 (1.9%)
Uniparental disomy	6 (5.7%)
Mosaic	1 (1.0%)
Unknown/not reported	19 (18.1%)
Ability for person with AS to be left alone, n (%)	
No, she/he needs continuous surveillance	101 (96.2%)
Yes, for less than one hour	4 (3.8%)
Yes, she/he can easily be left along for several hours (or more)	0 (0.0%)

*AS* Angelman syndrome

The majority of individuals with AS represented in the survey were children (17.1% were aged 18 +). Approximately half were male (49.5%), and the majority were white (85.7%). Nearly two-thirds received health insurance from Medicaid or the Children's Health Insurance Program (CHIP) (62.9%). Most survey responses indicated that individuals with AS had either a deletion (48.6%) or mutation (24.8%) genetic subtype, with nearly one quarter reporting that they did not know the AS genetic subtype (18.1%). Nearly all individuals with AS required continuous surveillance; none were able to be left alone for more than one hour at a time. Paid professional caregiving support was relied on by 30.5% of individuals.

### Annual economic impact

In total, the average annual economic impact of caregiving for persons with AS was estimated to be $79,837 (SD $55,505), with 37% of the total impact incurred through household costs to accommodate the individual with AS and 63% through caregiver employment-related impacts, lost caregiver leisure time, and additional caregiver healthcare costs.

#### Household costs to accommodate and care for individuals with AS

**Fig. 1:**
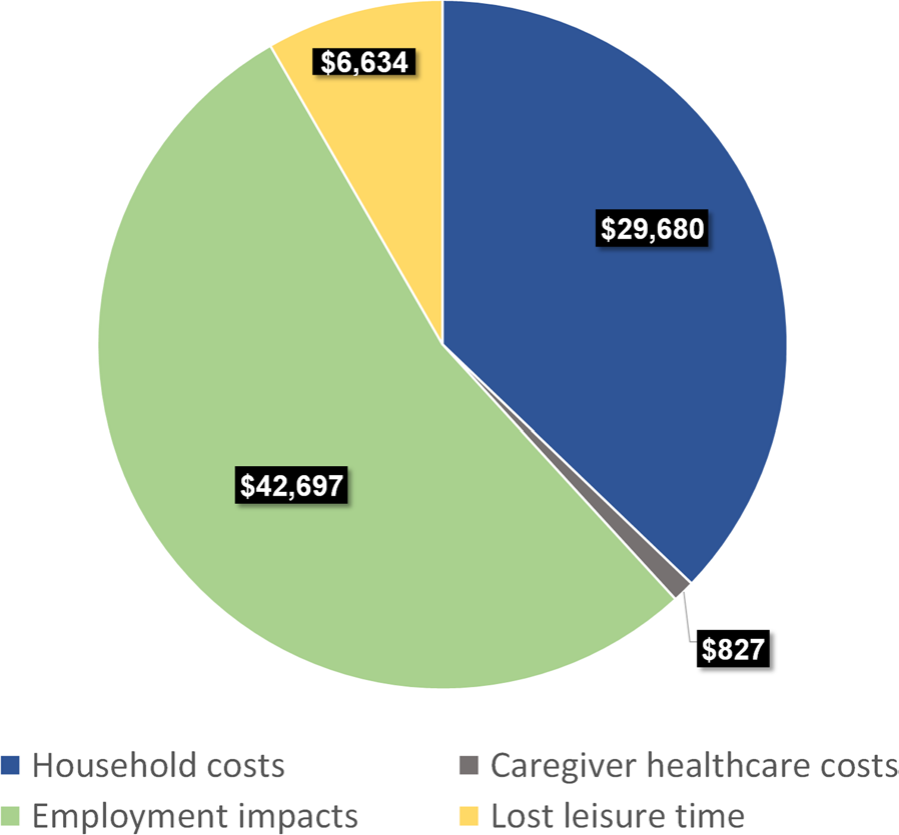
Average annual economic impact of caregiving for an individual with AS. AS, Angelman syndrome

On average, annual AS-related costs for goods and services incurred by households were $29,680 (SD $47,753) (Fig. 1; Supplementary Table 2, Additional File 1). Leading contributors were vehicle purchases or modifications (mean $6,717; SD $17,791), professional caregiving (mean $6,123; SD $17,335), home modifications or repairs (mean $4,387; SD $15,734), and supportive therapy (mean $3,269; SD $7,564). The most common AS-related vehicle-related expense in the past year was a new car or vehicle, reported by 19 (18.1%) caregivers (Supplementary Fig. 1, Additional File 1). Among these 19 caregivers, 58% also reported having purchased a wheelchair, stroller, walker, and/or other ambulatory aid in the past year. Professional caregiving largely consisted of at-home caregiving (e.g., personal health aid; 24.8%). Common home modifications or repairs included gates or fences (25.7%), installation of locks or stair locks (24.8%), bathroom modifications (21.9%), and home repairs due to damage or excess wear (21.0%). Supportive therapy commonly consisted of physical therapy (54.3%), speech therapy (51.4%), and occupational therapy (50.5%).

#### Caregiver healthcare costs

Annual healthcare costs incurred by caregivers as a result of their caregiving duties averaged $827 (SD $2,072) (Fig. 1; Supplementary Table 2, Additional File 1). Pharmaceutical and medical expenses were similar, with respective mean costs of $399 (SD $1,123) and $428 (SD $1,169) in the past 12 months.

#### Caregiver employment-related impact

The average annual impact of employment-related disruptions for caregivers was $42,697 (SD $28,309) (Fig. 1; Supplementary Table 2, Additional File 1). Annual impacts were highest from lost productivity during work hours (mean $19,536; SD $21,203), followed by impacts from stopping work (mean $15,387; SD $28,024) or reducing paid working hours due to caregiving demands (mean $7,774; SD $15,179)

The average degree of overall work impairment among caregivers was 62.3%, and the degree of daily activity impairment outside of work was 65.4% (Supplementary Fig. 2, Additional File 1). Most caregivers (83.8%) had experienced at least one employment-related disruption in their lifetime as a result of their caregiving duties (Supplementary Fig. 3, Additional File 1). In addition to reducing work hours (42.9%) and stopping work for more than one year (35.2%), common disruptions included being unable to find employment or take a job (24.8%) and changing to working what they considered to be unsociable hours (21.9%).

#### Caregiver leisure time impact

The average annual value of lost leisure time among caregivers was $6,634 (SD $4,652) (Fig. 1; Supplementary Table 2, Additional File 1). Many caregivers reported losing leisure time due to caregiving duties (87.6%).

### Caregiver quality of life

**Fig. 2:**
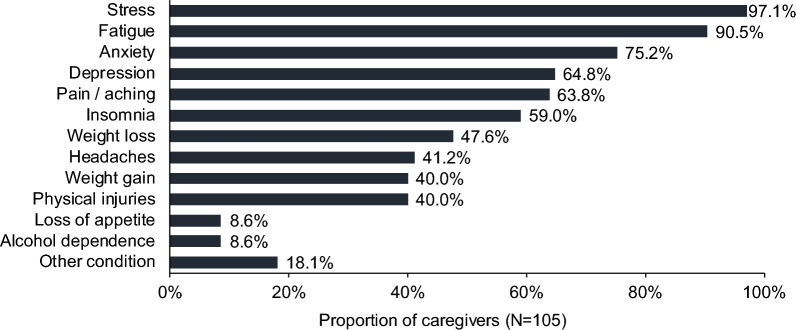
Health impacts associated with caregiving for an individual with AS. AS, Angelman syndrome

With respect to health impacts, nearly all caregivers reported experiencing stress (97.1%) or fatigue (90.5%) as a result of caring for a person with AS (Fig. 2). Most caregivers also experienced anxiety (75.2%), depression (64.8%), pain or aching (63.8%), and insomnia (59.0%). Weight loss (47.6%), headaches (41.2%), weight gain (40.0%), and physical injuries (40.0%) were also common.

Caregiver responses to the CarerQol quality-of-life self-assessment are summarized in Supplementary Fig. 4 Additional File 1. Nearly all (93.3%) caregivers reported having at least some fulfilment from carrying out care tasks, and most (82.9%) caregivers reported having at least some support carrying out care tasks. However, most reported having at least some problems combining care tasks with their own daily activities (84.7%), with their own physical health (83.8%), and with their own mental health (79.0%). Nearly two-thirds of caregivers (64.7%) reported having at least some financial problems as a result of their caregiving tasks and just under half (49.5%) reported having at least some relational problems with the care receiver. Across the survey sample, the average utility score accounting for all seven domains of the CarerQol was 65.1 (SD 20.0).

### Subgroup analysis

**Fig. 3:**
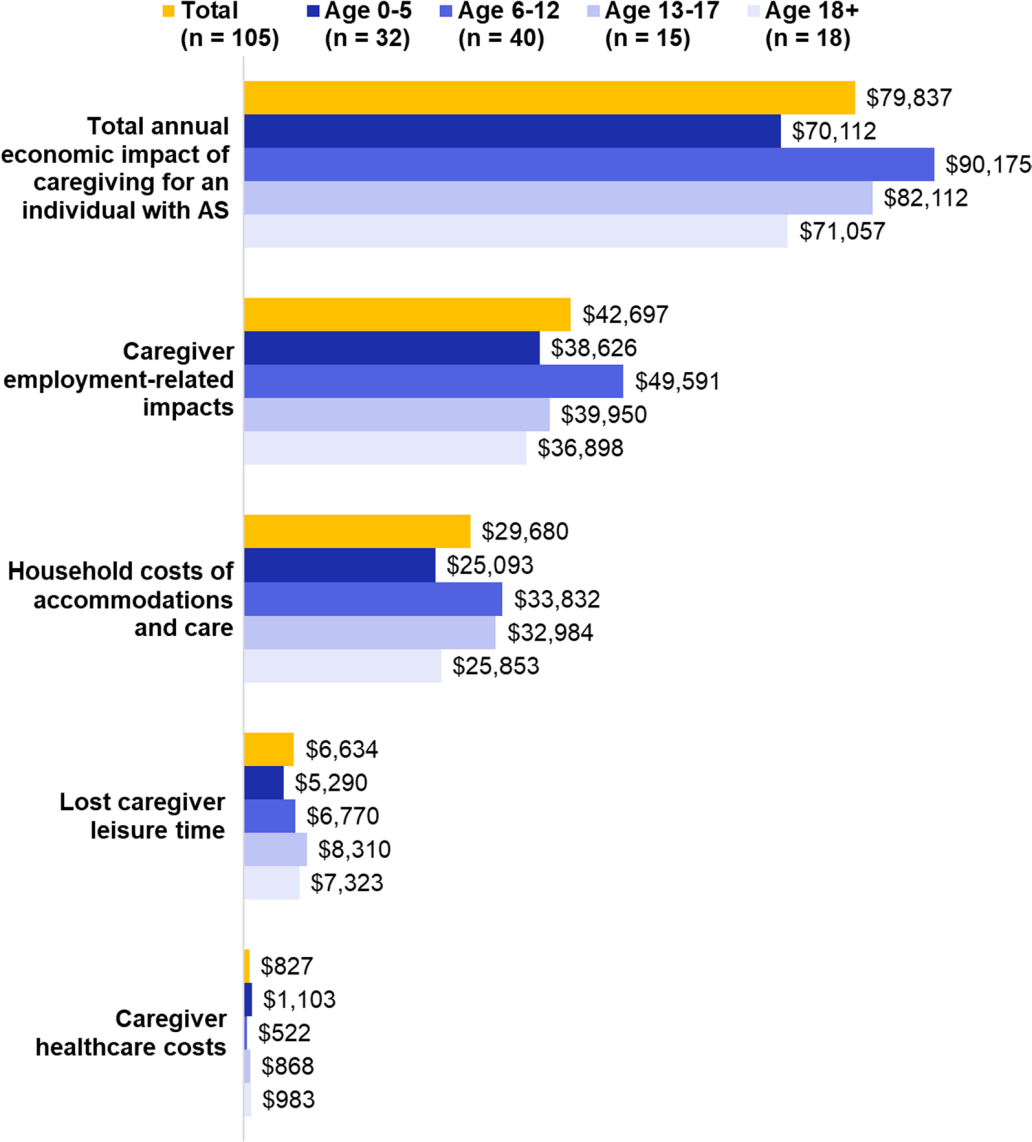
Average annual economic impact of caregiving, stratified by age of individuals with AS. AS, Angelman syndrome

The costs associated with caregiving for a person with AS were similar across age groups, with some variation observed across cost categories (Fig. 3). Average caregiver costs were highest among those caring for an individual age 6–12 (mean $90,715; SD $62,619) and lowest among those caring for an individual age 0–5 (mean $70,112; SD $49,411). This variation was largely attributable to differences in AS-related household costs and caregiver employment-related impacts.

## Discussion

This study provides evidence of the substantial economic impacts to caregivers arising from caregiving responsibilities for individuals with AS. Caregivers incur considerable costs to accommodate AS-related impairments and needs, particularly related to home- and vehicle-related expenses, professional caregiving, and supportive therapy. Additionally, caregivers experience substantial economic impacts in terms of lost work productivity and leisure time. These estimates reflect the extensive day-to-day needs of individuals with AS and the critical role that family caregivers play in accommodating these needs. As unpaid caregiving is often considered a ‘free’ source of care, the economic value of this care is often underestimated [29]. Notably, nearly 90% of caregivers surveyed in this study were female, and three-quarters identified themselves as the primary caregiver for an individual with AS.

To our knowledge, this is the first US-focused research study to quantify the economic impacts of caregiving for individuals with AS. Our findings are consistent with recent research on the economic impacts of caregiving in other rare disorders. For instance, the annual indirect and intangible costs of caring for individuals with Duchenne muscular dystrophy have been estimated to be in the range of $28,420 to $66,630 in 2014 USD, or roughly $37,000 to $87,000 in 2023 USD [28, 30, 31]. More recently, Yang et al. (2022) estimated the total indirect costs of rare diseases in the US, including caregiver work productivity loss and non-medical household expenditures [16]. Among caregivers for individuals age < 18 representing twenty different chromosomal abnormalities, average employment-related impacts were estimated to be $30,482 (2019 USD) and household expenditures were estimated to be $19,626 (2019 USD) [16, 30]. Our estimate of economic impacts for employment-related impacts (mean $42,697; SD $28,309) and household expenditures (mean $29,680; SD $47,753) for caregivers of individuals with AS are comparable but higher, which may reflect the substantial levels of family and caregiver support needs for individuals with AS.

Our findings are subject to certain limitations. First, the cross-sectional design of the survey instrument and absence of a control group limits the ability to attribute all caregiver impacts to AS rather than other factors. Additionally, all data collected through retrospective surveys are subject to participants’ recall bias. For instance, caregivers may have been more likely to remember large expenses but not remember smaller ones. Relatedly, because respondents were asked only about costs incurred in the past twelve months, infrequent major expenses (e.g., vehicle purchases, home repairs or renovations) may not be comprehensively captured.

In addition, our findings may not be generalizable to all caregivers or families of individuals with AS in the US. Families that engage with patient advocacy groups may have more resources or opportunities with respect to seeking aid or navigating AS-related challenges. Over 80% of caregivers who completed this survey were White, over 70% had a Bachelor’s or postgraduate degree, and more than 60% reported household earnings equal to or greater than the national median household income ($74,580 as of 2022) [32]. As a result, there may be differences in the challenges and costs experienced by survey respondents compared to other caregivers. Moreover, work productivity, leisure time, and quality of life impacts were only captured for one caregiver in a given household. Therefore, total household impacts may be understated. Similarly, our findings may not be generalizable to the experiences of male caregivers, secondary caregivers, or siblings. Future research could assess the broader impacts on these populations. Finally, differences in state-level health programs, health services, and price levels for household costs may vary across US states and impact the overall economic burden faced by caregivers. Information on state of residence was not captured in the current survey, and differences in the economic impact of caregiving across US states may be an important area for future research.

Our findings may also not be generalizable to families caring for individuals with AS who differ from the 105 individuals with AS represented in our study sample. There is a lack of nationally representative data regarding the sociodemographic characteristics of individuals with AS, making it difficult to determine the representativeness of our sample. Similar age and sex distributions were observed in our sample as compared to a cohort of 302 persons with AS represented in the AS Natural History Study (ASNHS) [33]. However, only 17.1% of individuals with AS in the present study sample were 18 years of age or older, which may limit the representativeness of findings for caregivers of adult individuals with AS. Compared to caregivers for pediatric and adolescent individuals with AS, caregivers for adult individuals may experience different economic impacts due to distinct care needs. Compared to the ASNHS, our study also had a lower rate of confirmed individuals with a deletion subtype (48.6% versus 70% in ASNHS), which has been associated with more severe functional impairments [33]. As such, the impacts associated with caregiving for AS may be higher among a broader population of individuals with AS.

Finally, the present study was solely focused on quantifying AS-related economic impacts for US caregivers of individuals with AS. However, a full assessment of the economic impacts from AS in the United States would need to account for a broader set of perspectives across multiple stakeholders, including health insurance payers, government representatives at both the Federal and state level, and individuals with AS. Assessments for these stakeholders may provide further insight into categories of economic impact not well-covered in this study, such as complete costs for adult individuals with AS who require long-term care. As this research focused on eliciting costs borne specifically by caregivers, other methods and data sources may be better suited to capturing these broader impacts. In sum, findings from our research should not be considered a complete assessment of economic impacts due to AS in the United States. Future research focused on these additional perspectives is warranted to gain a comprehensive understanding of the full economic impact from AS.

## Conclusions

This study quantified the considerable economic impacts to caregivers of individuals with AS, with an average impact of $79,837 per year. The largest contributor was employment-related impacts, followed by household expenditures on goods and services to accommodate individuals with AS. Reducing economic and quality of life impacts borne by caregivers may also have important consequences for clinical well-being among individuals with AS, as they are highly care-dependent throughout their lifetimes. This study’s findings may be utilized in future research to better estimate value derived from therapeutic advances in AS, and to direct resources toward mitigating these economic impacts for households.

## Supplementary Information

Additional file1

## Data Availability

Survey data generated during this study are not available due to participant privacy protections. Researchers can request access to the Global Angelman Syndrome Registry data through the registry website (https://www.angelmanregistry.info/data-access/).

## Abbreviations

AAC: Augmentative and alternative communication
ABA: Applied behavior analysis
AS: Angelman syndrome
ASF: Angelman Syndrome Foundation
ASNHS: AS Natural History Study
BiPAP: Bilevel positive airway pressure
CarerQol: The Care-related Quality of Life instrument
CHIP: Children's Health Insurance Program
CPAP: Continuous positive airway pressure
FAST: Foundation for Angelman Therapeutics
G-tube: Gastronomy tube
GASR: Global Angelman Syndrome Registry
iMTA: Institute for Medical Technology Assessment
iVICQ: IMTA Valuation of Informal Care Questionnaire
NG tube: Nasogastric tube
SD: Standard deviation
UBE3A: Ubiquitin-protein ligase E3A
US: United States
VA: Veterans Affairs
WPAI: Work Productivity and Activity Impairment
