# Supplementary material for: The economic impact of caregiving for individuals with Angelman syndrome in the United States: results from a caregiver survey
Source: Orphanet J Rare Dis. 2025 Feb 21;20:82. doi: 10.1186/s13023-025-03551-4 (PMC11846284; doi:10.1186/s13023-025-03551-4)
Supplement: Supplementary file 1 — Additional file1 [file 13023_2025_3551_MOESM1_ESM.docx]

Additional File 1. Supplemental information

Methods

**Annual economic impact of caregiving for an individual with AS**

#### Household costs to accommodate and care for individuals with AS

#### Household costs included expenses in the past twelve months for the following categories/items:

| **Category** | **Item** |
| --- | --- |
| Home modifications / repairs | Gates / fences |
|  | Installed locks or stair locks |
|  | Made bathroom modifications |
|  | Home repairs due to damage or excess wear |
|  | Installed alerting devices |
|  | Made kitchen modifications |
|  | Installed railings |
|  | Installed ramps or street level entrances |
|  | Installed automatic or easy to open doors |
|  | Expanded home to accommodate caregivers |
|  | Widened doorways or hallways |
|  | Created accessible parking or drop-off site |
|  | Other home modifications |
| Vehicle purchase / modifications | Purchasing a new car / vehicle |
|  | Special car seat |
|  | Installing air conditioning |
|  | Creating a large trunk or storage area |
|  | Installing a button that opens the car door |
|  | Other vehicle purchase / modification |
| Medical equipment purchases | AAC device(s) |
|  | Walker / leg braces |
|  | Stroller |
|  | Activity / bath chair(s) |
|  | Safety bed(s) |
|  | Toilet aid |
|  | Sensory adaptation aids |
|  | Ambulatory aids |
|  | Wheelchair |
|  | Helmet |
|  | Personal location monitoring system |
|  | Feeding tube (e.g., NG tube, G-tube) |
|  | Seizure monitors |
|  | Respiratory equipment (e.g., CPAP/BiPaP, tracheotomies, pulmonary vests) |
|  | Other medical equipment |
| Long-term care | Special day care |
|  | Skilled nursing facility |
|  | Assisted living facility |
|  | Group home / foster home |
|  | Other long-term care |
| Professional caregiving | At-home caregiver (e.g., personal home care aid / companion / respite care) |
|  | Home health aid / personal care assistant |
|  | Certified nursing assistant |
|  | Skilled nurse |
|  | Other caregiving service |
| Supportive therapy | Physical therapy |
|  | Speech therapy |
|  | Occupational therapy |
|  | Early childhood intervention |
|  | Horse therapy |
|  | Behavioral therapy / ABA |
|  | Hydrotherapy |
|  | Music therapy |
|  | Neurofeedback therapy |
|  | Other supportive therapy |
| School / education | Assistive school equipment |
|  | Tuition fees for person with AS |
|  | Shared one-on-one aide in school |
|  | Teacher aide training |
|  | Private one-on-one aide in school |
|  | Educational tutoring |
|  | Other school / special education expenses |
| Healthcare resources | Prescription drugs |
|  | Primary care doctor office or home visits |
|  | Outpatient visits with an adult or pediatric specialist |
|  | Emergency room visits |
|  | Hospital admissions |
|  | Outpatient surgery or day surgery |
|  | Ambulance trips (911) |
|  | Other |
| Additional out-of-pocket expenses | Toys to keep individual relaxed / calm (not including communication devices) |
|  | Incontinence products (e.g., briefs, pull-ups, diapers) |
|  | Non-pharmaceutical supplements / vitamins (e.g., melatonin, multivitamin) |
|  | Special food / meals (e.g., formula) |
|  | Special clothing (e.g., for body temperature regulation, G-tube accessible clothing, bibs) |
|  | Administrative / legal fees |
|  | Other expenses |

*AAC* Augmentative and alternative communication; *ABA* Applied Behavior Analysis; *BiPAP* bilevel positive airway pressure; *CPAP* continuous positive airway pressure; *G-tube* gastronomy tube; *NG* nasogastric tube.

For each household cost category, caregivers could select a range that corresponded to their costs (e.g., $500 to < $1,000) or enter a specific numerical value. For the purpose of calculating mean (SD) costs, if the caregiver selected a range, the cost was assumed to be the midpoint of the selected range. For example, if a caregiver selected ‘$500 to < $1,000’ for the ‘Vehicle purchase / modifications’ category, their cost was assumed to be $750 for that category.

For each cost category, the mean (SD) cost across caregivers was calculated among all caregivers in the study sample. A cost of $0 was assigned to those who did not report an expense. Additionally, conditional mean (SD) costs were calculated for each expense category; only caregivers who reported having an expense were included in the calculation.

Caregivers were asked to provide their best estimate of the costs that were specifically paid by their households (e.g., credit card, cash, family savings), not including any costs paid by external sources (e.g., health insurance, government, or charitable organizations). Caregivers were asked to provide their best estimate of costs that were incurred as a direct result of the care recipient’s AS.

#### Caregiver healthcare costs associated with care for individuals with AS

#### Healthcare costs included costs related to pharmaceutical treatments and medical care related to AS. Specifically, pharmaceutical expenses included expenses in the past twelve months for any medications for pain, headaches, insomnia, anxiety, depression, or other physical, mental, or emotional health due to caregiving for the person with AS. Medical expenses included expenses in the past twelve months for any medical care from healthcare professionals for managing pain, headaches, insomnia, anxiety, depression, or other physical, mental, or emotional health due to caring for the person with AS.

For each cost category, the mean (SD) cost across caregivers was calculated among all caregivers in the study sample. A cost of $0 was assigned to those who did not report an expense for the category.

*Lost caregiver work productivity associated with care for individuals with AS*

Work productivity was evaluated using questions from the caregiver version of the WPAI questionnaire ([1](#_ENREF_1)). To assess the value of productivity loss because the caregiver stopped working: caregivers who reported a current employment status of ‘Unemployed,’ ‘Retired,’ ‘Homemaker,’ or ‘Other’ were asked if they stopped working due to caregiving for a person with AS and, if so, how many hours they typically worked before they stopped working. The annual economic value of lost productivity was calculated by multiplying the mean (SD) hours worked per week before work was stopped by the mean hourly wage in the US ($33.74 as of July 2023) ([2](#_ENREF_2)) and then multiplying by 52 weeks per year.

To assess the value of productivity loss because the caregiver’s working hours were reduced: caregivers who reported a current employment status of ‘Working full time for pay’ or ‘Working part time for pay’ were asked how many hours they currently worked for pay per week, whether they reduced their paid working hours to care for the person with AS, and if so, how many hours they typically worked per week prior to reducing their hours. Hours lost because of work hour reductions were estimated as the difference in hours before work was reduced and hours after work was reduced. The economic value of lost work productivity was calculated by multiplying the mean (SD) hours of lost productivity per week by the mean hourly wage in the US and then multiplying by 52 weeks per year.

To assess the value of productivity loss because the caregiver’s work productivity decreased: caregivers who reported a current employment status of ‘Working full time for pay’ or ‘Working part time for pay’ were asked how much caregiving for a person with AS affected their productivity while working. The response was converted to a percentage and multiplied by their reported paid work hours during the past week to estimate hours of lost productivity. The value of lost productivity was calculated by multiplying the mean (SD) hours of lost productivity per week by the mean hourly wage in the US and then multiplying by 52 weeks per year.

*Lost caregiver leisure time associated with care for individuals with AS*

Caregivers were asked whether they had given up time spent on leisure in order to provide informal care to the person with AS and, if so, how many hours per week they had given up. The value of lost leisure time was calculated by multiplying the mean (SD) value of lost leisure time by the mean hourly wage in the US and then multiplying by 52 weeks per year, and finally multiplying by an adjustment factor of 35% ([3](#_ENREF_3)).

**Additional descriptive measures of caregiver impacts**

*Average degree of work and activity impairment per the WPAI*

Questions were derived from the caregiver WPAI questionnaire ([1](#_ENREF_1)). Caregivers who reported a current employment status of ‘Working full time for pay’ or ‘Working part time for pay’ were asked about work and non-work activity in the past week in order to calculate the percentage degree of impairment they experienced as a result of caregiving for a person with AS, as follows:

| Absenteeism | If they had missed paid working hours due to caregiving for a person with AS, and if so, by how much. Work hours missed due to caregiving for a person with AS were divided by the total contracted hours (sum of hours missed and hours worked) |
| --- | --- |
| Presenteeism | Extent to which caregiving for a person with AS affected productivity while working, on a scale from 1 to 10, converted to a percentage |
| Overall work impairment | The sum of paid working hours impacted by absenteeism or presenteeism, as a percentage of total typical paid working hours |
| Activity impairment | How much caregiving for a person with AS affected their ability to do regular daily activities other than work, such as work around the house, shopping, childcare, exercise, or studying – on a scale from 1 to 10 converted to a percentage |

The mean (SD) degree of overall work impairment was calculated across all employed caregivers. The mean (SD) degree of activity impairment was calculated across all caregivers.

*Employment disruptions*

Caregivers were also asked whether they or another household member had ever experienced employment disruptions as a result of caregiving for a person with AS.

*Health impacts*

To further characterize health-related quality of life impacts, caregivers were asked whether they had ever experienced or developed a set of specific health conditions or symptoms as a result of caregiving for a person with AS. The list of health conditions was derived based on input from caregivers of individuals with AS consulted during survey development.

*Caregiver quality of life*

Caregivers were asked to review seven ‘I’ statements and assess the degree to which each statement reflected their situation at the time of the survey. Statements included in the self-assessment were derived from the Care-related Quality of Life instrument (CarerQol), an instrument that is included as part of the iMTA Valuation of Informal Care Questionnaire (iVICQ). For each statement in the CarerQol, respondents were asked to score each statement at three answering levels regarding problems/support/fulfilment: 1) “No”, 2) “Some”, 3) “A lot”. Aggregate responses to these seven statements were descriptively summarized as percentages. Separately, a utility score was generated for each respondent based on their responses to these seven statements. For each caring state (represented by the unique combination of responses to the seven statements), a utility score was previously valued from the Dutch general population, with a value set ranging from 0 (worst caring situation) to 100 (best caring situation) ([4](#_ENREF_4)). These utility scores were summarized using means and standard deviations across the survey sample.

Results

Supplementary Table 1a. Additional caregiver demographics

| **Demographic variables** | | **N = 105** |
| --- | --- | --- |
| *Marital status, n (%)* | |  |
|  | Married or living with someone | 90 (85.7%) |
|  | Divorced or separated | 10 (9.5%) |
|  | Never married | 5 (4.8%) |
| *Hours currently worked per week, n (%)* | |  |
|  | 10 | 5 (4.8%) |
|  | 20 | 11 (10.5%) |
|  | 30 | 9 (8.6%) |
|  | 40 | 35 (33.3%) |
|  | Other/Not employed | 45 (42.9%) |
| *Number of people in household working for pay in past 12 months, n (%)* | |  |
|  | 0 | 7 (6.7%) |
|  | 1 | 33 (31.4%) |
|  | 2 | 50 (47.6%) |
|  | 3 | 3 (2.9%) |
|  | 4+ | 3 (2.9%) |
|  | Not reported | 9 (8.6%) |
| *Household wage/salary earnings in the last 12 months, n (%)^a^* | |  |
|  | < $10,000 | 6 (5.7%) |
|  | $10,000 to < $25,000 | 0 (0.0%) |
|  | $25,000 to < $50,000 | 8 (7.6%) |
|  | $50,000 to < $75,000 | 12 (11.4%) |
|  | $75,000 to < $100,000 | 11 (10.5%) |
|  | $100,000 to < $125,000 | 12 (11.4%) |
|  | $125,000 to < $150,000 | 6 (5.7%) |
|  | $150,000 to < $175,000 | 8 (7.6%) |
|  | $175,000 to < $200,000 | 4 (3.8%) |
|  | $200,000 to < $250,000 | 10 (9.5%) |
|  | $250,000 to < $300,000 | 6 (5.7%) |
|  | $300,000 to < $350,000 | 0 (0.0%) |
|  | $350,000 or more | 8 (7.6%) |
|  | Not reported | 14 (13.3%) |
| **Caregiving-related variables** | |  |
| Years spent providing informal care to the person with AS, n (%) | |  |
|  | Less than 1 year | 8 (7.6%) |
|  | 1 to < 2 years | 5 (4.8%) |
|  | 2 to < 3 years | 10 (9.5%) |
|  | 3 to < 5 years | 14 (13.3%) |
|  | 5 to < 10 years | 31 (29.5%) |
|  | 10 to < 15 years | 11 (10.5%) |
|  | 15 to < 20 years | 14 (13.3%) |
|  | 20 years or more | 12 (11.4%) |
| *AS* Angelman syndrome; *SD* standard deviation   1. Not including any income from self-employment. | | |

Supplementary Table 1b. Additional demographic and clinical characteristics of individuals with AS

| **Demographic variables** | | **N = 105** |
| --- | --- | --- |
| *Insurance coverage status during the past 12 months, n (%)* | |  |
|  | Medical and pharmacy coverage | 97 (92.4%) |
|  | Medical coverage only | 4 (3.8%) |
|  | Pharmacy coverage only | 1 (1.0%) |
|  | Neither medical nor pharmacy coverage | 3 (2.9%) |
| *Type(s) of insurance coverage during the past 12 months, n (%)* | |  |
|  | Commercial insurance through employer | 66 (62.9%) |
|  | Other commercial insurance | 4 (3.8%) |
|  | Medicaid or Children's Health Insurance Program (CHIP) | 66 (62.9%) |
|  | Medicare or Medicare supplemental | 11 (10.5%) |
|  | Military (e.g., TRICARE, VA) | 3 (2.9%) |
|  | Uninsured | 0 (0.0%) |
|  | Other | 6 (5.7%) |
|  | Not applicable | 1 (1.0%) |
| *Current work situation* | |  |
|  | Not employed | 103 (98.1%) |
|  | Employed in mainstream or supported employment | 2 (1.9%) |
| *Personal wage/salary earnings in the last 12 months, n (%)^a^* | |  |
|  | < $10,000 | 2 (100.0%) |
| *Other sources of direct financial assistance for person with AS, n (%)* | |  |
|  | Social Security | 17 (16.2%) |
|  | Social Security Disability Insurance | 10 (9.5%) |
|  | Supplemental Security Income | 16 (15.2%) |
|  | Other state or local public assistance or welfare payments | 2 (1.9%) |
|  | Any other sources of income received regularly | 2 (1.9%) |
| **Clinical and caregiving-related variables** | |  |
| *Current living situation, n (%)* | |  |
|  | Living in family household | 101 (96.2%) |
|  | Living in a residential or nursing home | 2 (1.9%) |
|  | Sharing a household with at least one other person | 2 (1.9%) |
| *Hours per day of care received in the past week, n (%)^b^* | |  |
|  | < 4 | 0 (0.0%) |
|  | 4-8 | 0 (0.0%) |
|  | 8-12 | 1 (1.0%) |
|  | 12-16 | 6 (5.7%) |
|  | 16-20 | 19 (18.1%) |
|  | 20-24 | 12 (11.4%) |
|  | 24 | 58 (55.2%) |
|  | Not reported | 9 (8.6%) |
| *Professional caregiving needs* | |  |
|  | Person with AS needs additional care beyond respondent, n (%) | 32 (30.5%) |
|  | Additional care hours needed, mean (SD)^c^ | 45.2 (45.1) |
| *AS* Angelman syndrome; *CHIP* Children's Health Insurance Program; *SD* standard deviation; *VA* Veterans Affairs   1. Among individuals with AS who were employed. 2. Includes time being present in the home alongside the individual with AS. 3. Among individuals with AS needing care beyond the respondent. | | |

Supplementary Table 2. Detailed annual costs of caregiving for an individual with AS

|  | **Caregivers impacted, n (%)** | **Mean (SD) cost** |
| --- | --- | --- |
| **Household costs of accommodations and care^a^** |  | **$29680 ($47753)** |
| Home modifications / repairs | 43 (41.0%) | $4387 ($15734) |
| Vehicle purchase / modifications | 23 (21.9%) | $6717 ($17791) |
| Medical equipment purchases | 53 (50.5%) | $1372 ($3394) |
| Long-term care | 3 (2.9%) | $2857 ($21738) |
| Professional caregiving | 32 (30.5%) | $6123 ($17335) |
| Supportive therapy | 66 (62.9%) | $3269 ($7564) |
| School / education expenses | 31 (29.5%) | $677 ($2034) |
| Healthcare resource use | 61 (58.1%) | $1057 ($2273) |
| Additional expenses | 57 (54.3%) | $3220 ($5607) |
|  |  |  |
| **Caregiver healthcare costs^a^** |  | **$827 ($2072)** |
| Pharmaceutical expenses | 57 (54.3%) | $399 ($1123) |
| Medical expenses | 56 (53.3%) | $428 ($1169) |
|  |  |  |
| **Lost caregiver work productivity** |  | **$42697 ($28309)** |
| Stopped working | 26 (24.8%) | $15387 ($28024) |
| Reduced paid working hours | 28 (26.7%) | $7774 ($15179) |
| Reduced work productivity | 61 (58.1%) | $19536 ($21203) |
|  |  |  |
| **Lost caregiver leisure time** | 92 (87.6%) | **$6634 ($4652)** |
|  |  |  |
| ***Total annual costs of caregiving for an individual with AS*** |  | ***$79837 ($55505)*** |

*AS* Angelman syndrome; *SD* standard deviation

^a^ Caregivers were asked to provide their best estimate of the costs that were specifically paid by their households (e.g., credit card, cash, family savings), not including any costs paid by external sources (e.g., health insurance, government, or charitable organizations).

Supplementary Table 3. Conditional annual household costs of accommodations and care

| **Household costs of accommodations and care^a^** | **Caregivers reporting expenses, n (%)** | **Mean (SD) cost among those with expenses** |
| --- | --- | --- |
| Home modifications / repairs | 43 (41.0%) | $10712 ($23317) |
| Vehicle purchase / modifications | 23 (21.9%) | $30665 ($26993) |
| Medical equipment purchases | 53 (50.5%) | $2718 ($4394) |
| Long-term care | 3 (2.9%) | $100000 ($100000) |
| Professional caregiving | 32 (30.5%) | $20092 ($26804) |
| Supportive therapy | 66 (62.9%) | $5201 ($9019) |
| School / education expenses | 31 (29.5%) | $2293 ($3242) |
| Healthcare resource use | 61 (58.1%) | $1819 ($2747) |
| Additional expenses | 57 (54.3%) | $5932 ($6482) |

*AS* Angelman syndrome; *SD* standard deviation

^a^ Caregivers were asked to provide their best estimate of the costs that were specifically paid by their households (e.g., credit card, cash, family savings), not including any costs paid by external sources (e.g., health insurance, government, or charitable organizations).

Supplementary Figure 1. Proportion of caregivers reporting past-year AS-related household expenses

*AAC* Augmentative and alternative communication; *ABA* Applied Behavior Analysis; *BiPAP* bilevel positive airway pressure; *CPAP* continuous positive airway pressure; *G-tube* gastronomy tube; *NG* nasogastric tube.

Supplementary Figure 2. Work productivity and activity impairment (WPAI) among caregivers


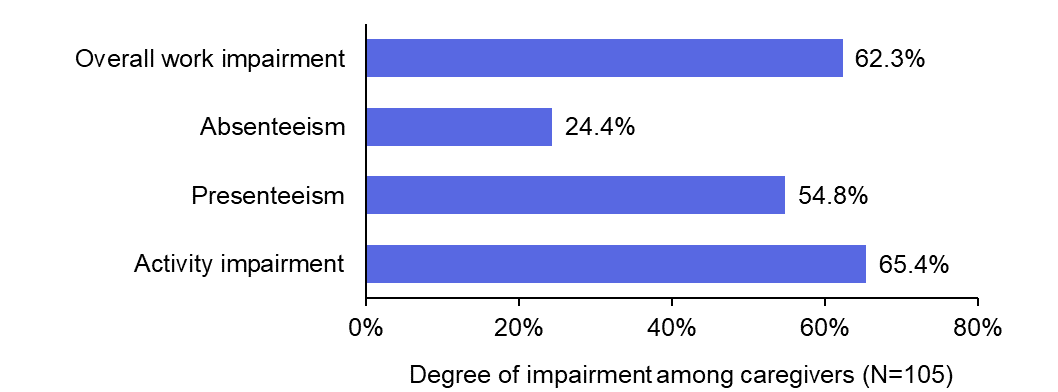


Degree of impairment for “Overall work impairment”, “Absenteeism”, and “Presenteeism” reported for the subset of caregivers who self-reported as employed for the WPAI questionnaire (N=61).

Supplementary Figure 3. Employment disruptions associated with caregiving for an individual with AS

Supplementary Figure 4. Self-assessment of caregiving situation

Responses sum to 99% rather than 100% for two statements (‘I have financial problems because of my care tasks’ and ‘I have problems combining my care tasks with my own daily activities’) for which one caregiver did not provide a response.

References

1. Giovannetti ER, Wolff JL, Frick KD, Boult C. Construct validity of the Work Productivity and Activity Impairment questionnaire across informal caregivers of chronically ill older patients. Value Health. 2009;12(6):1011-7.

2. United States Department of Labor BoLS. July 2023 Employment Situation. August 4, 2023.

3. Landfeldt E, Lindgren P, Bell CF, Schmitt C, Guglieri M, Straub V, et al. The burden of Duchenne muscular dystrophy: an international, cross-sectional study. Neurology. 2014;83(6):529-36.

4. Hoefman R, Van Exel N, Rose J, van de Wetering E, Brouwer W. A discrete choice experiment to obtain a tariff for valuing informal care situations measured with the CarerQol instrument. Med Decis Making. 2014;34(1):84-96.
